# Supplementary material for: The role of intraoperative microelectrode recording and stimulation in subthalamic lead placement for Parkinson’s disease
Source: PLoS One. 2020 Nov 6;15(11):e0241752. doi: 10.1371/journal.pone.0241752 (PMC7647091; doi:10.1371/journal.pone.0241752)
Supplement: Attachment — Submitted filename: Reviewer Letter.docx [file pone.0241752.s001.docx]

Dear Editors and Reviewers,

thank you for critically reviewing our manuscript "The role of intraoperative microelectrode recording and stimulation in subthalamic lead placement for Parkinson’s disease". We are grateful for the valuable reviewer comments. We revised the manuscript under consideration of all reviewer comments and addressed all reviewers’ requests.

**Comments to the Author**

1. Is the manuscript technically sound, and do the data support the conclusions?
The manuscript must describe a technically sound piece of scientific research with data that supports the conclusions. Experiments must have been conducted rigorously, with appropriate controls, replication, and sample sizes. The conclusions must be drawn appropriately based on the data presented.

Reviewer #1: Yes

2. Has the statistical analysis been performed appropriately and rigorously?

Reviewer #1: I Don't Know

A big part of statistical analysis in this manuscript consists of descriptive statistics. We used t-test for the evaluation of differences between the patient group with decisions made in accordance with MER compared to the group where the decisions were made according to the test stimulations and not to the MER. We stated this in the chapter “Statistical analysis” of the methods section of the manuscript.

3. Have the authors made all data underlying the findings in their manuscript fully available?
The [PLOS Data policy](http://www.plosone.org/static/policies.action#sharing) requires authors to make all data underlying the findings described in their manuscript fully available without restriction, with rare exception (please refer to the Data Availability Statement in the manuscript PDF file). The data should be provided as part of the manuscript or its supporting information or deposited to a public repository. For example, in addition to summary statistics, the data points behind means, medians and variance measures should be available. If there are restrictions on publicly sharing data—e.g. participant privacy or use of data from a third party—those must be specified.

Reviewer #1: No

We provided a PDF-file including all data underlying the findings in this manuscript.

4. Is the manuscript presented in an intelligible fashion and written in standard English?
PLOS ONE does not copyedit accepted manuscripts, so the language in submitted articles must be clear, correct, and unambiguous. Any typographical or grammatical errors should be corrected at revision, so please note any specific errors here.

Reviewer #1: Yes

5. Review Comments to the Author

Reviewer #1: The findings from this report are valuable to publish especially since this technique is being used less frequently although I do not think they are breaking any new ground here. Awake versus asleep DBS surgery for unclear reasons has been pushed to the center as highly relevant to outcomes. It may be that both approaches are safe and effective.
I did not see the data sheet attached to the PDF. The authors should include this to comply with PLOS ONE.

We agree with the reviewer that probably both approaches are safe and effective. However, we consider it as an important objective to evaluate the impact of intraoperative MER during awake DBS surgery on the long-term motor outcome of the patients. From patients’ perspective asleep DBS procedure is the more comfortable and therefore, preferable option, compared to awake procedure. Hence, providing evidence towards comparable results of both awake and asleep DBS surgery would facilitate the clinical decision-making in this field.

We provided a PDF-file including all data underlying the findings in this manuscript.

Best regards

Vesna Malinova
